# The Role of *SLC22A1* and Genomic Ancestry on Toxicity during Treatment in Children with Acute Lymphoblastic Leukemia of the Amazon Region

**DOI:** 10.3390/genes13040610

**Published:** 2022-03-29

**Authors:** Sweny de S. M. Fernandes, Luciana P. C. Leitão, Amanda de N. Cohen-Paes, Laura P. A. Gellen, Lucas F. Pastana, Darlen C. de Carvalho, Antônio A. C. Modesto, Ana C. A. da Costa, Alayde V. Wanderley, Carlos H. V. de Lima, Esdras E. B. Pereira, Marianne R. Fernandes, Rommel M. R. Burbano, Paulo P. de Assumpção, Sidney E. B. dos Santos, Ney P. C. dos Santos

**Affiliations:** 1 Oncology Research Center, Federal University of Pará, Belém 66073, PA, Brazil; swenymf@gmail.com (S.d.S.M.F.); colaresluciana@gmail.com (L.P.C.L.); acohencastro@gmail.com (A.d.N.C.-P.); laura.patricia.agellen@hotmail.com (L.P.A.G.); lucas.pastana@ics.ufpa.br (L.F.P.); darlen.c.carvalho@gmail.com (D.C.d.C.); antonioacm@ufpa.br (A.A.C.M.); carolinecostaodonto19@gmail.com (A.C.A.d.C.); alaydevieira@yahoo.com.br (A.V.W.); carloshvl@hotmail.com (C.H.V.d.L.); rommelburbano@gmail.com (R.M.R.B.); assumpcaopp@gmail.com (P.P.d.A.); sidneysantos@ufpa.br (S.E.B.d.S.); npcsantos.ufpa@gmail.com (N.P.C.d.S.); 2 Laboratory of Human and Medical Genetics, Institute of Biological Science, Federal University of Pará, Belém 66077-830, PA, Brazil; esdrasedgarbp@gmail.com

**Keywords:** acute lymphoid leukemia, severe toxicity, ancestry, *NUDT1*, SLC22A1

## Abstract

In Brazil, Acute lymphoid leukemia (ALL) is the leading cause of cancer deaths in children and adolescents. Treatment toxicity is one of the reasons for stopping chemotherapy. Amerindian genomic ancestry is an important factor for this event due to fluctuations in frequencies of genetic variants, as in the *NUDT15* and *SLC22A1* genes, which make up the pharmacokinetic and pharmacodynamic pathways of chemotherapy. This study aimed to investigate possible associations between *NUDT15* (rs1272632214) and *SLC22A1* (rs202220802) gene polymorphism and genomic ancestry as a risk of treatment toxicities in patients with childhood ALL in the Amazon region of Brazil. The studied population consisted of 51 patients with a recent diagnosis of ALL when experiencing induction therapy relative to the BFM 2009 protocol. Our results evidenced a significant association of risk of severe infectious toxicity for the variant of the *SLC22A1* gene (OR: 3.18, *p* = 0.031). Genetic ancestry analyses demonstrated that patients who had a high contribution of African ancestry had a significant protective effect for the development of toxicity (OR: 0.174; *p* = 0.010), possibly due to risk effects of the Amerindian contribution. Our results indicate that mixed populations with a high degree of African ancestry have a lower risk of developing general toxicity during induction therapy for ALL. In addition, individuals with the *SLC22A1* variant have a higher risk of developing severe infectious toxicity while undergoing the same therapy.

## 1. Introduction

Representing 75% of acute leukemias and approximately 35% of all malignant neoplasms in childhood, ALL is a type of cancer that mostly affects children worldwide [1,2]. In Brazil, this type of leukemia is the leading cause of cancer deaths in children and adolescents aged 0–19 years [3]. The survival rate of ALL is approximately 80% when using multiagent chemotherapy regimens [4]. Despite this, treatment toxicity is one of the main reasons for interrupting or discontinuing chemotherapy, which can affect the quality of life of patients not only during but also after its completion [1,5].

An investigation carried out in a miscegenated population with high ancestry of Amazonian Amerindians, treated with the protocol of European Group Berlin-Frankfurt-Münster (BFM) for ALL, showed that 65.3% of this population presents grade 3 and 4 toxicity [6], a frequency higher than that found in other world populations submitted to the same protocol (26%) [7]. Thus, Amerindian genomic ancestry could be an important factor for the high rates of toxicity reported in the Amazon region in patients undergoing treatment for ALL. Other studies have shown even worse survival rates among Hispanic ALL patients when compared to Europeans, Americans, or Asians [8]. Therefore, the different drug responses related to ethnic differences can be explained, in part, by fluctuations in the frequencies of important functional gene variants that make up the pharmacokinetic and pharmacodynamic pathway of chemotherapeutic agents [9,10,11].

Thus, a number of studies have been looking for genetic markers that can help adjust doses of anti-leukemic drugs in order to optimize clinical outcomes and avoid the occurrence of toxicities [12,13,14]. Some of these genes are present in the Absorption, Distribution, Metabolism, and Excretion (ADME) pathways of drugs, such as NUDT15 and the SLAC22A1 transporter. The mutational frequency of the *NUDT15* gene varies widely among different continental populations. In Northern Brazil, a study reported that *NUDT15* variants alter the metabolization profile of drugs used in the standard ALL protocol [15]. In the case of the *SLC22A1* gene, it is also known as organic cationic transporter 1 (*OCT1*, encoded by the *SLC22A1* gene) and is part of a family that plays an important role in drug–drug interactions (DDI); several polymorphisms of this gene have been associated with alterations in drug availability, response, and toxicity [16].

The aim of this study was to investigate possible associations between NUDT15 (rs1272632214) and SLC22A1 (rs202220802) gene polymorphism and genomic ancestry as a risk of treatment toxicities in patients with childhood ALL in the Amazon region of Brazil.

## 2. Methods

### 2.1. Ethical Aspects

The protocol used in this study was approved by the Research Ethics Committee of the Health Sciences Institute of the Federal University of Pará, under protocol number 119.649/2012. Individuals under 18 years of age and their guardians were duly informed about the research. Volunteers agreed to participate in the study by signing the Free and Informed Assent Term and their guardians signed the Free and Informed Consent Term.

### 2.2. Study Populations

This is a prospective study, which included 51 patients with a recent diagnosis of ALL by immunophenotyping and treated at a referral center for pediatric cancer treatment (Otávio Lobo Hospital), in Belém of Pará, in the Amazon Region of Brazil. The patients included in the research were between 1 and 18 years of age; had nonrecurrence; were without comorbidities or other types of cancer; and had morphological, immunophenotypic, and, when available, molecular diagnoses. All patients who did not meet this inclusion criteria were excluded from the study.

### 2.3. Induction Therapy Protocol for ALL

Included patients underwent induction therapy for ALL with the BFM 2009 protocol [17]. During induction therapy, which lasts for a total of 64 days, patients undergo phase 1 corticosteroids for 36 days; four doses of vincristine; 2 doses of doxorubicin (in low-risk and intermediate-risk patients) or 4 doses of doxorubicin (in high-risk patients); and l-asparaginase (8 doses), and during the second phase of cyclophosphamide induction (two doses), patients received 16 doses of cytarabine and 28 consecutive days of mercaptopurine [17].

### 2.4. Assessment and Classification of Toxicity

Laboratory tests, including transaminases and blood count, were computed in a table at three times during the induction phase: on the first day of treatment, prior to the use of any medication; before the second induction phase, before the use of mercaptopurine but as a patient was already sensitized to other drugs; and after the second induction phase. Adverse events such as anorexia, colitis, diarrhea, dyspepsia, mucositis, nausea, vomiting, neutropenia, and documented or undocumented infection were computed, and numerical stratification was applied to events according to the CTC-NCI guide (Common Toxicity Criteria). After collecting information, adverse effects were classified according to the degree, by considering CTC-NCI: mild/moderate (number 0, 1, and 2) and severe (3 and 4) [18].

### 2.5. DNA Extraction and Quantification

DNA was extracted by the conventional method with phenol-chloroform according to Sambrook [19]. Samples were quantified in the NanoDrop ND-1000 equipment (Thermo Scientific NanoDrop Products, Wilmington, DE, USA).

### 2.6. Selection of Polymorphisms

Potential genetic markers were selected from a previous survey in the literature [15,16]. Thus, we obtained 2 polymorphisms, *SLC22A1* (rs202220802) and *NUDT15* (rs1272632214), related to susceptibility, toxicity, and response in the treatment of ALL.

### 2.7. Genotyping of Polymorphisms

Genotyping of single nucleotide polymorphisms was performed by allelic discrimination using the TaqMan OpenArray Genotyping technology, in the QuantStudio™ 12K Flex Real-Time PCR System (Applied Biosystems, Life Technologies, Carlsbad, CA, USA) according to the protocol recommended by Applied Biosystem. The TaqMan Genotyper software was used to analyze plaque data and genotype reading accuracy, in addition to genotyping quality control.

### 2.8. Genomic Ancestry 

Analysis was performed using a panel of 61 autosomal informative ancestry markers according to Ramos et al. [20]. Two multiplex PCRs were performed, followed by electrophoresis in the sequencer in ABI Prism 3130 (Applied Biosystems, Life Technologies, Carlsbad, CA, USA) and analysis in the GeneMapper ID v.3.2 program (Applied Biosystems, Life Technologies, Carlsbad, CA, USA). The individual proportions of European, African, and Amerindian genetic ancestry were estimated using Structure v.2.3.3 software.

### 2.9. Statistical Analysis

A descriptive analysis of the data referring to the characterization of the sample was carried out. Quantitative variables were first submitted to the Kolmogorov–Smirnov test to analyze the distribution of normality. The individual proportions of European, African, and Amerindian genetic ancestry were estimated using Structure 2.3.3 software.

For the comparative analysis between the study groups regarding the variables for characterizing the samples, the Chi-square test was applied for categorical variables and the Mann–Whitney test for continuous variables. In order to analyze the association of *SLC22A1* and *NUDT15* gene polymorphisms with the risk of general toxicity in ALL, logistic regression was performed, controlled by the African ancestry variable. In order to analyze the association of *SLC22A1* and *NUDT15* gene polymorphisms with the risk of severe infectious toxicity in ALL, an age-controlled logistic regression was performed.

All statistical analyses were performed using the statistical package of the SPSS 20.0 software while respecting the significance level of 5% (*p* value ≤ 0.05).

## 3. Results

In the present study, overall toxicity occurred in 47.1% (95%CI: 33.3–62.7) among ALL patients. In the analysis of demographic and epidemiological characteristics, it can be observed that the groups were similar in terms of gender, age, and ALL subtype. However, they differed significantly in the distribution of African ancestry. These results suggested a loss in the contribution of African ancestry (*p* value: 0.029) in the toxic group compared to the nontoxic group (Table 1).

Regarding the type of toxicity observed, 62.9% of patients with general toxicity had symptoms of gastrointestinal toxicity, 96.2% had liver toxicity, 88.8% were classified as moderate or severe hepatotoxicity, 92.5% had infectious toxicity, and 53.8% were classified as severe infectious toxicity (Table 1).

The occurrence of deaths among the investigated patients was 29.4% (95%CI: 17.6–41.2%). Regarding the occurrence of deaths due to general toxicity between the groups of ALL patients, it can be observed that there was no significant difference in the occurrence of deaths between the groups (*p* value: 0.375) (Table 1).

**Figure 1:**
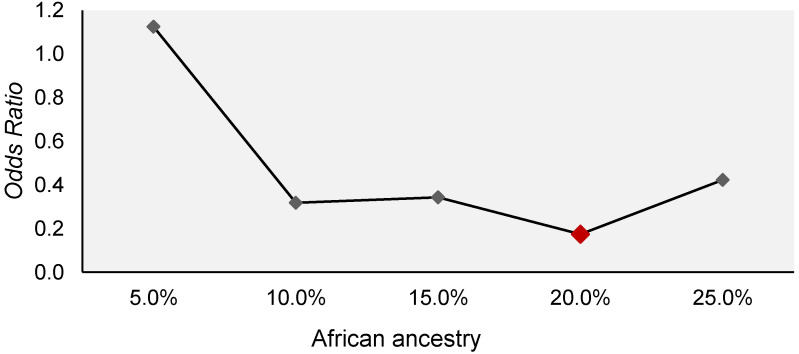
Variation in odds ratios recorded for different percentages of African ancestry as a protective factor for general toxicity in patients with ALL of the Amazon Region.

The influence of the distribution of African ancestry on the susceptibility to the development of general toxicity in patients with ALL was evaluated using a logistic regression model (Figure 1). The analysis showed that African ancestry in the range between 10% and 25% demonstrated protection for the development of general toxicity in patients with ALL. However, only a 20% contribution showed a significant protective effect, 0.17 (OR: 0.174; 95%CI: 0.04–0.65; *p* value: 0.010).

**Figure 2:**
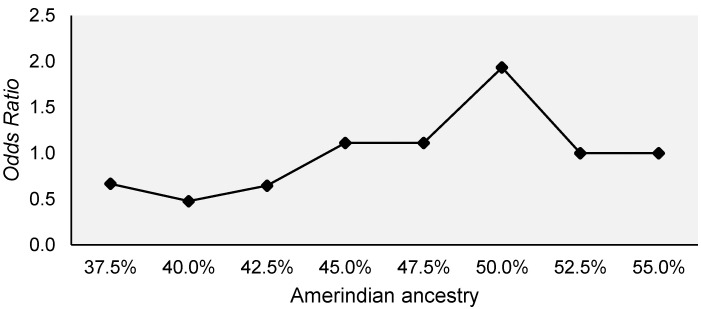
Variation in the odds ratios recorded for different percentages of Amerindian ancestry as a risk factor for general toxicity in patients with ALL of the Amazon Region.

This protective effect of African ancestry may have occurred in counterpoint to the risk effect shown by the Amerindian contribution, as the increase in African ancestry is offset by Amerindian ancestry, and vice versa. Logistic regression model analysis did not reveal a significant effect on overall toxicity in ALL patients. However, the analysis allowed us to observe a potential risk effect of Amerindian ancestry in the range between 45% and 55% for general toxicity in patients with ALL, reaching the peak risk in 50% of this ancestry (OR: 1.93; 95%CI: 0.30–12.16; *p* value: 0.483) (Figure 2).

The influence of gene variants SLC22A1 (rs202220802) and NUDT15 (rs1272632214) for the development of general toxicity in patients with ALL was evaluated using the multivariate logistic regression model, controlled by African ancestry. This analysis showed that none of the genotypes and none of the alleles of both polymorphisms showed significant effects on the occurrence of general toxicity in patients with ALL (Table 2).

The influence of gene variants SLC22A1 (rs202220802) and NUDT15 (rs1272632214) on the development of severe infectious toxicity in patients with ALL was also evaluated using the age-controlled logistic regression model. This analysis showed that, for the NUDT gene variant (rs1272632214), none of the genotypes and none of the alleles showed significant effects on the occurrence of severe infectious toxicity in patients with ALL (Table 3). However, for the SLC gene variant (rs202220802), it was possible to observe that homozygous and heterozygous genotypes with deletions were more frequent in the group with severe infectious toxicity. In addition, the deletion allele was also significantly more frequent in the group with severe infectious toxicity, demonstrating a risk effect for this event and increasing the chance of its occurrence by about three times (OR: 3.18; 95%CI: 1.11–9.11, *p* value: 0.031) (Table 3).

## 4. Discussion

Currently, studies on new treatments for ALL are not only aimed at discovering more efficient antineoplastics but also seek to minimize the toxic effects of these medications. Given the intensification of treatment in the last three decades, the chance of death related to the toxicity of the therapy can be compared to chance of relapse in low-risk patients [21]. There are several symptoms that can occur during ALL treatment associated with the unwanted pharmacological effect of the medications. In the literature, the occurrence of mainly gastrointestinal and hematological effects during treatment for ALL is evidenced and is mainly associated with genetic variations in genes such as *NUDT15*, *TPMT*, and *PDE4D* causing more severe toxicities in certain ethnic groups such as the Amerindians [15,22,23].

Infections and febrile neutropenia, which are changes resulting from hematological toxicity and which are considered infectious toxicities, can impact the response and effectiveness of treatment. The results are in accordance with the data found in the literature, which describes an increase in hematological events associated with the genetic variability of certain populations [24,25,26,27].

Genetic variants in *NUDT15*, which characterize a loss or severe decrease in its function, can lead patients treated with thiopurines to an excessive activation of these drugs, causing serious adverse effects, such as hematopoietic toxicities in malignant conditions such as ALL [8]. These variants are already present in the guidelines of the Consortium for the Implementation of Clinical Pharmacogenetics (CPIC) as an alternative to prevent toxicity in these patients [28]. A study recently published by our research group demonstrated that the NUDT15*2 and NUDT15*4 haplotypes of *NUDT15* are present in high frequencies in Amerindian and mixed populations of Northern Brazil when compared to other continental populations, which implies the alteration of the metabolization profile of these individuals when treated with the standard regimen for ALL [15]. In this study, however, no association of the *NUDT15* gene with any of the toxicities investigated here was found.

*SCL22A1* gene encodes hOCT1 (Human Organic Cation Transporter 1), a genetically variable transporter that is strongly expressed in the epithelial barriers and sinusoidal membrane of the human liver [29]. hOCT1 plays a role in the pharmacodynamics and pharmacokinetics of anticancer, antiviral, anti-inflammatory, and antiemetic drugs, as well as drugs used in the treatment of neurological diseases [29,30]. Variants that cause complete loss of hOCT1 activity have already been identified, and on average 1 in 11 Europeans or Americans have a reduction in hepatic drug absorption because they have poor hOCT1 transporters [31,32,33]. Studies carried out on the treatment of different types of leukemias showed results indicating that variants in the *SLC22A1* gene have different treatment toxicities or clinical responses [29,34]. Our study demonstrated that the rs202220802 polymorphism of the *SLC22A1* gene, with the deletion allele being a risk factor, had approximately three times the risk of suffering severe hematotoxicity (OR: 3.18; 95%CI: 1.11–9.11, *p* value: 0.031).

Our results showed a protective factor for toxicity to ALL treatment in patients above 20% of African ancestry. A study by Yao et al. [35] also pointed to a reduction in the risk of toxicities related to the treatment of ALL in patients aged 1–18 years who had a high degree of African genomic ancestry, particularly related to fractures and osteonecrosis. This same study presented data relating the worst treatment outcomes to the high degree of Native American ancestry, which has already been described in different studies [8,11] and which are associated with our findings, further demonstrating a potential risk for the development of severe treatment toxicities in patients with a range of up to 50% of Amerindian ancestry. The degree of Amerindian ancestry associated with the potential risk of developing toxicities may be related to the genomic ancestral opposition to African ancestry, which confers a potential for protection. However, we cannot exclude the possibility that environmental, sociocultural, and dietary differences play a role in the observed toxicities.

The important role of ancestry and polymorphism in the *SLC22A1* gene in toxicity that occurs in the treatment of ALL was evidenced in the present study. However, more studies are needed to minimize some of the limitations presented, such as the increase in participants; the inclusion of other clinical factors; sociocultural and nutritional particularities that may be related to toxicity; and addressing other metabolic pathways that are possibly associated with toxicity in the treatment of ALL.

## 5. Conclusions

In conclusion, the *SLC22A1* gene variant (rs202220802) was associated with a potential risk of developing severe infectious toxicity in patients with ALL. In addition, African ancestry demonstrated protection for the development of general toxicity in patients with ALL. These results are important for stimulating new genomic studies that can identify genetic variants with high frequency or those that are exclusive to populations with high miscegenation degrees that can explain the predisposition of these patients to severe toxicities.

## Figures and Tables

**Table 1:** Demographic and epidemiological characteristics, according to the occurrence of general toxicity, of patients with ALL of the Amazon Region.

Variables	General Toxicity		*p* Value
	Yes (n. 27)	No (n. 24)	
**Gender**			
Female	9 (33.3%)	13 (54.2%)	0.134 ^a^
Male	18 (66.7%)	11 (45.8%)	
**Age (years)**			
Average (±SD)	7.19 (±4.34)	6.96 (±4.43)	0.776 ^b^
**ALL Sub-type**			
ALL B	23 (37.0%)	19 (16.7%)	0.804 ^a^
ALL T	4 (14.8%)	5 (20.8%)	
**Ancestry**			
European	0.494 (±0.520)	0.496 (±0.120)	0.439 ^b^
Amerindian	0.342 (±0.149)	0.324 (±0.123)	0.970 ^b^
African	0.143 (±0.060)	0.179 (±0.057)	0.029 ^b,^*
**Toxicity type**			
**Gastrointestinal**			
Low	9 (33.3%)	NA	NA
Moderate	8 (29.6%)	NA	
High	0 (0.0%)	NA	
**Hepatic**			
Low	2 (7.4%)	NA	NA
Moderate	21 (77.7%)	NA	
High	3 (11.1%)	NA	
**Infectious**			
Low	0 (0.0%)	NA	NA
Moderate	11 (40.7%)	NA	
High	14 (51.8%)	NA	
**Mortality**			
Yes	6 (22.2%)	9 (37.5%)	0.375 ^a^
No	21 (77.8%)	15 (62.5%)	

ALL, acute lymphoid leukemia. SD, standard deviation. ^a^ Chi-square test. ^b^ Mann–Whitney test. * *p* value ≤ 0.05. NA, not applicable.

**Table 2:** Comparative analysis of the variation in polymorphisms of the *SLC22A1* (rs202220802) and *NUDT15* (rs1272632214) genes, by the occurrence of general toxicity, in patients with ALL of the Amazon Region.

Polymorphisms	General Toxicity		*p* Value ^a^	*p* Value ^b^	OR (95%IC)
	Yes (n. 27)	No (n. 24)			
***SLC22A1***					
n-n	5 (18.5%)	9 (37.5%)	0.112	0.230	0.43 (0.11–1.69)
n-ins	6 (22.2%)	2 (8.3%)		0.209	3.64 (0.51–20.63)
n-del	9 (33.3%)	6 (25.0%)		0.435	1.70 (0.45–6.51)
del-ins	1 (3.7%)	5 (20.8%)		0.072	0.12 (0.01–1.20)
del-del	5 (18.5%)	1 (4.2%)		0.196	4.68 (0.45–48.62)
ins-ins	1 (3.7%)	1 (4.2%)		0.635	0.50 (0.03–8.77)
Allele n	25 (46.3%)	26 (54.2%)	0.578	0.737	0.86 (0.37–2.01)
Allele ins	9 (16.7%)	9 (18.8%)		0.502	0.68 (2.31–2.05)
Allele del	20 (37.0%)	13 (27.1%)		0.366	1.52 (0.61–3.77)
***NUDT15***					
Del-Del	20 (76.9%)	22 (91.7%)	0.155	0.321	0.39 (0.06–2.64)
Ins-Del	6 (23.1%)	2 (8.3%)			
Allele Del	46 (88.5%)	46 (95.8%)	0.175	0.347	2.32 (0.40–13.55)
Allele Ins	6 (11.5%)	2 (4.2%)			

^a^ Chi-square test. ^b^ Multivariate logistic regression adjusts for African ancestry. OR, odds ratio. CI, confidence interval.

**Table 3:** Comparative analysis of the variation in polymorphisms of the *SLC22A1* (rs202220802) and *NUDT15* (rs1272632214) genes, by the occurrence of severe infectious toxicity in patients with ALL of the Amazon Region.

Polymorphisms	Severe Infectious Toxicity		*p* Value ^a^	*p* Value ^b^	OR (95%IC)
	Yes (n. 14)	No (n. 37)			
***SLC22A1***					
n-n	3 (21.4%)	11 (29.7%)	0.304	0.597	0.66 (0.15–3.02)
n-ins	2 (14.3%)	6 (16.2%)		0.216	0.27 (0.03–2.13)
n-del	6 (42.9%)	9 (24.3%)		0.194	2.80 (0.69–11.32)
del-ins	0 (0.0%)	6 (16.2%)		0.999	0.00 (0.00–0.00)
del-del	3 (21.4%)	3 (8.1%)		0.085	6.17 (0.78–49.11)
ins-ins	0 (0.0%)	2 (5.4%)		0.999	0.00 (0.00–0.00)
Allele n	14 (50.0%)	37 (50.0%)	0.155	0.715	1.19 (1.06–1.33)
Allele ins	2 (7.1%)	16 (21.6%)		0.049*	0.19 (0.04–0.99)
Allele del	12 (42.9%)	21 (28.4%)		0.031*	3.18 (1.11–9.11)
***NUDT15***					
Del-Del	10 (76.9%)	32 (86.5%)	0.418	0.333	0.43 (0.08–2.33)
Ins-Del	3 (23.1%)	5 (13.5%)			
Allele Del	23 (88.5%)	69 (93.2%)	0.432	0.356	2.11 (0.43–10.35)
Allele Ins	3 (11.5%)	5 (6.8%)			

^a^ Chi-square test. ^b^ Age-adjusted multivariate logistic regression. OR, odds ratio. IC, confidence interval. * *p*-value ≤ 0.05.

## Data Availability

Not applicable.
